# Women’s experiences of accessing vaccines during pregnancy and for their babies during COVID-19: Helen Skirrow

**DOI:** 10.1093/eurpub/ckac131.057

**Published:** 2022-10-25

**Authors:** H Skirrow, S Barnett, S Bell, S Mounier-Jack, B Kampmann, B Holder

**Affiliations:** 1 Department of Primary Care and Public Health, Imperial College London, London, UK; 2 Institute of Reproductive and Developmental Biology, Imperial College London, London, UK; 3 Department of Global Health and Development, LSHTM, London, UK; 4 The Vaccine Centre, LSHTM, London, UK; 5 Vaccines and Immunity Theme, MRC Unit The Gambia at LSHTM, Banjul, Gambia

## Abstract

**Background:**

COVID-19 changed access to healthcare, including vaccinations, in the United Kingdom (UK). This study explored UK women’s experiences of accessing pertussis vaccination during pregnancy and infant vaccinations during COVID-19.

**Methods:**

An online cross-sectional survey was completed, between 3rd August-11th October 2020, by 1404 women aged 16+ years who were pregnant at some point after the first UK lockdown from March 23rd 2020. Ten follow-up semi-structured interviews were conducted.

**Results:**

Most women surveyed were pregnant (65.7%) and a third postnatal (34.3%). Almost all women (95.6%) were aware that pertussis vaccination is recommended in pregnancy. Most pregnant (72.1%) and postnatal women (84.0%) had received pertussis vaccination however, access issues were reported. Over a third (39.6%) of women had a pregnancy vaccination appointment changed. COVID-19 made it physically difficult to access pregnancy vaccinations for one fifth (21.5%) of women and physically difficult to access infant vaccinations for almost half of women (45.8%). Nearly half of women (45.2%) reported feeling less safe attending pregnancy vaccinations and over three quarters (76.3%) less safe attending infant vaccinations due to COVID-19. The majority (94.2%) felt it was important to get their baby vaccinated during COVID-19. Pregnant women from ethnic-minorities and lower-income households were less likely to have been vaccinated. Minority-ethnicity women were more likely to report access problems and feeling less safe attending vaccinations for both themselves and their babies. Qualitative analysis found women experienced difficulties accessing antenatal care and relied on knowledge from previous pregnancies to access vaccine appointments.

**Conclusions:**

COVID-19 disrupted access to vaccinations in the UK. Vaccine services must ensure equitable access to vaccine appointments during ongoing and future pandemics including tailoring services for lower income and ethnic minority families.

**Key messages:**

• Pregnancy and infant vaccines was disrupted by COVID-19 with women feeling less safe and having difficulties accessing vaccinations with ethnic minority women more likely to report access issues.

• Equitable access to routine pregnancy and infant vaccine appointments must be prioritised during future pandemics, including considering tailoring services for different population groups.

